# Correlation between the Mandibular Lingula Position and Some Anatomical Landmarks in Cone Beam CT

**DOI:** 10.3390/healthcare9121747

**Published:** 2021-12-17

**Authors:** Saturnino Marco Lupi, Jessica Landini, Giorgia Olivieri, Claudia Todaro, Andrea Scribante, Ruggero Rodriguez y Baena

**Affiliations:** School of Dentistry, Department of Clinical Surgical, Diagnostic and Pediatric Sciences, University of Pavia, 27100 Pavia, Italy; jessica.landini01@universitadipavia.it (J.L.); giorgiaolivieri96@gmail.com (G.O.); claudia.todaro01@universitadipavia.it (C.T.); andrea.scribante@unipv.it (A.S.); ruggero.rodriguez@unipv.it (R.R.y.B.)

**Keywords:** mandibular nerve, anesthesia, orthognathic surgery

## Abstract

Background: the position of the mandibular lingula (Li) affects the success rate of the inferior alveolar nerve block (IANB) and ramus osteotomies. This study evaluated the position of the Li, to investigate the anatomical relationship between the Li and some anatomical measurements using cone beam computed tomography (CBCT). Methods: 201 hemimandibular CBCTs of 111 patients (43 males and 68 females; 18 to 88 years old) were retrospectively evaluated. The Li location was determined from the lingula tip to: the occlusal plane, the anterior and posterior borders of the mandibular ramus, the lower border of the mandible, the distal surface of the mandibular second molar, and the mandibular notch. We evaluated the correlations between the Li and the anteroposterior diameter of the mandibular ramus; the vertical distance between condyle and mandibular angle; the mesial–distal diameter of the first, second, and third mandibular molar, the intercanine distance, the intermolar distances among the first, second, and third mandibular molars; the distance between the intermolar line of the first molar and midline, and the length of the mandibular body. Results: the vertical distance of the Li from the occlusal plane was 11.22 ± 4.27 mm. Some parameters significantly correlated with the anatomical measurements taken into consideration. Conclusions: the present study provides new information concerning the Li and mandibular anatomy in the Italian population. Moreover, by correlating some anatomic measurements to the Li position, the localization of the Li is made possible, indirectly through the measurement of some distances between anatomical landmarks.

## 1. Introduction

The lingula (Li) is a tongue-shaped bony prominence located on the medial surface of the mandibular ramus; it overlaps the mandibular foramen (MF). It is attached to the sphenomandibular ligament and can sometimes be palpated through the oral mucosa [1].

Li was first described by Johann Baptist Ritter von Spix in 1815, and for this reason, it was named ‘‘Spix’s ossicle” or “Spix’s spine” [2].

Because of its relation to nervous and vascular structures, since the inferior alveolar nerve (IAN) enters the MF at the Li position, understanding the structures related to the Li position is crucial. The study of the Li features provides significant information related to oral and maxillofacial surgical procedures, such as sagittal split ramus osteotomy (SSRO), intraoral vertical ramus osteotomy (IVRO) [3], inverted-L osteotomy [4], orthognathic surgery, mandibular trauma management, eradication of benign and malignant lesions, pre-prosthetic surgery [5], cyst removals in mandibular ramus, temporomandibular, and joint reconstruction [5]. If oral and maxillofacial surgeons do not identify the lingula correctly, intraoperative complications, such as bleeding, unfavorable fractures, and nerve injuries, could occur [6]. 

Furthermore, the presence of anatomical variables in the position of the lingula must be considered in the planning of surgical interventions, due to its clinical relevance [7,8]. The lingula is also used to locate the site for the injection of local anesthetics during IAN block anesthesia: the anesthetic must be injected in the pterygomandibular space near the lingula and the MF of the mandible [9]. The failure rate of the IAN block was reported to range between 5 and 35%, even when performed by dentists who have several years of experience [10]. The most common reason for this failure points to the inaccurate placement of the hypodermic needle tip, which is not close enough to the MF [11]. Variability in the location of the MF and the Li contribute to the low success rate. Inadequate knowledge of the anatomy of the medial aspect of the ramus may result in failure of the mandibular block or, more importantly, in complications, such as nerve trauma, vascular injury, and intravascular, intraglandular, or intramuscular injection [12]. Determining the precise anatomical locations of the Li is essential in order to achieve an effective anesthesia of the IAN, and to obtain a favorable fracture line of the mandibular ramus, preventing IAN damage and other complications during orthognathic surgery. 

Several studies investigated the anatomy and the location of the Li using dry skulls [4,13,14,15], panoramic radiographs [5,16,17], or conventional computed tomography (CT) [18]. Panoramic radiography provides different magnifications of the maxilla and the mandible, causing difficulties in visualizing mandibular landmarks [19]; moreover, panoramic radiography involves an inevitable distortion linked to the positioning of the investigated structures on different planes [20,21].

In contrast, three-dimensional (3D) reconstructed images from cone beam computed tomography (CBCT), which is commonly used in dental practice, are more reliable and accurate than 2D radiography of craniofacial structures, because CBCT can produce highly accurate images and provide precise locations of anatomical structures [22]. The application of CBCT in dentistry has rapidly developed in recent years, especially in implantology, because CBCT has overcome many disadvantages of CT, such as the high dose of radiation, long radiation exposure time, and low resolution ratio [23]. The aim of the present study was to locate the lingula in relation to several anatomical landmarks and to evaluate the correlation between the distance between some anatomical points and the position of the lingula in the Italian population using CBCT. Ultimately, the purpose of this study is to provide anatomical information on the lingula position, relevant for the IAN block and orthognathic surgery. 

## 2. Materials and Methods

In this retrospective, analytical study, 201 sides of hemimandibular CBCT reconstructions, relative to patients treated at the Dental Clinic, Department of Clinical Surgical, Diagnostic and Pediatric Sciences of the University of Pavia, between November 2017 and March 2021, were analyzed. All patients requested CBCT examinations for clinical diagnoses and treatment planning, including oral surgery, dental implants, orthodontics, endodontics, and oral pathology.

The CBCT radiographs were taken with a Scanora® multimodal radiography system (Soredex; Tuusula, Finland) operating with voltage settings ranging from 66  to 70 kVp; the field of view was 75 x 145 mm when scanning the entire mandible and 75 x 100 mm when scanning the hemimandible. The inclusion criteria were: male/female aged 18 years or older, good quality of the CBCT mandibular images, absence of any pathological conditions or deformities in the jaws. 

**Figure 1:**
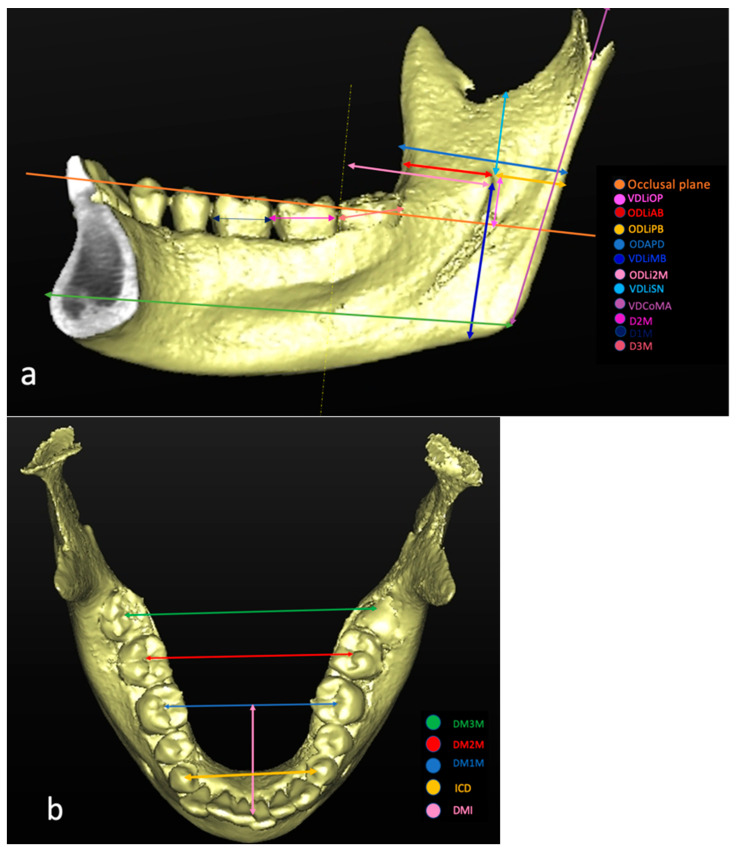
Measurements taken on the 3D reconstruction: (**a**) lateral and (**b**) occlusal views.

The exclusion criteria were: inadequate CBCT image quality (patient movement, operator errors, CBCT scans that did not include the mandible); history of trauma or orthognathic surgery; presence of pathologic bone disease or unerupted teeth in a related region; syndromic patients; severe atrophy of the lower jaw; pathological conditions or deformities in the jaw; presence of metallic material capable of producing artifacts. The original sample group consisted of 396 sides of hemimandibular CBCTs relative to 218 patients. As per the study selection criteria, the final sample group included 201 CBCT reconstructions relative to 111 patients (68 females and 43 males), 18 to 88 years old. All images were assessed in a dark room on a 27-inch monitor (5120-by-2880 resolution, iMac, Apple Inc., Cupertino, CA, USA) by a single examiner with more than 15 years of experience in analyzing CBCT. Given the large number of data to be collected and CBCT to be examined, data collection took place in multiple sessions, lasting approximately 2 hours. To evaluate the intraexaminer agreement, the measurements were repeated three times in 10% of the sample, and at least one month after the previous one. Using the CBCT data, 3D models of the mandibles were constructed using SimPlant Pro 18® software (Dentsply, Hasselt, Belgium) with a voxel size of 0.2 mm and slice thickness of 0.2 mm. To determine the exact location of the Li in each CBCT reconstruction, the measures listed in Table 1 were considered (Figure 1).

The distances between anatomical landmarks to be correlated with the positions of the lingula are listed in Table 2 (Figure 1).

The point of the reference for the mandibular lingula, in all measurements, was its tip (the highest point of the lingula). The measurements were taken after tracing the joining lines between the pre-established landmarks on the planes passing through Spix’s spine perpendicular and parallel to the occlusal plane.

In order to locate the apex of Spix’s spine, a 3D reconstruction of the CBCT image was performed (Figure 1). The occlusal plane (OP) was defined using three points: mesiobuccal cusp tips of both mandibular first molars and a mid-point of both mandibular central incisors as the horizontal reference plane [23,24]. In order to obtain the distance between Spix’s spine and the OP, an adaptation of the axial plane to the OP was carried out [24,25] and then the distance between the tip of the lingula and the axial plane was measured in the coronal plane previously corrected [25].

The horizontal distance from the anterior margin of the mandibular branch to Spix’s spine (ODLiAB), as well as the distance from the posterior margin of the mandibular branch to Spix’s spine (ODLiPB), was obtained using the most anterior and posterior points of the mandibular branch on the axial plane passing through Spix’s spine and parallel to the OP.

The vertical distance between the lower mandibular margin and Spix’s spine (VDLiMB) was measured on the frontal plane passing through the apex of Spix’s spine as the distance from the apex of the Li to the lowest point of the mandible [3,6,26,27].

The distance from the most distal point of the crown of the second molar to Spix’s spine (ODLi2M) was measured in the plane parallel to the OP passing through Spix’s spine from the orthogonal projection of the most distal point of the crown to Spix’s spine [15].

The vertical distance between the sigmoid notch and Spix’s spine (VDLiSN) was measured on the sagittal plane from the lowest point of the sigmoid notch to the lingular tip [6,23,25,28]. The vertical distance from the condyle to the mandibular angle (VDCoMA) was measured on the sagittal plane as the distance from the highest point of the mandibular condyle to the gonion.

The mesiodistal diameter of the molars (D1M, D2M, D3M) was measured on the sagittal plane following the definition by Moorrees and Reed, according to which, the diameter is the largest dimension of the crown in the mesial distal direction, parallel to the occlusal surface [29]. The intercanine distance (ICD) was obtained on the frontal plane and measured as the distance from the cusps of the lower right to the left canines.

The intermolar distance between molars (DM1M, DM2M, and DM3M) was measured on the frontal plane using the central fossae of the molars as landmarks.

The molar–midline distance (DMLM) was measured on the axial plane as the space between the midline passing through the lower incisors and the intermolar line 36–46.

The length of the mandibular body (LMAN) was measured on the sagittal plane between the most-posterior point of the mandible and the pogonion.

To reduce bias that may occur in setting the reference point and measuring the distances, the CBCT analyses of all patients were performed by the same researcher. Once the measurements were taken, a descriptive statistical analysis was carried out.

Evaluations were performed on the entire study sample and on samples stratified by gender.

The minimum and maximum values of each measurement were also reported.

Intraexaminer agreement was evaluated by ICC. ICC estimates and their 95% confident intervals were calculated using R 4.0.4 (The Foundation for Statistical Computing, http://www.R-project.org) based on a mean-rating (k = 3), absolute-agreement, 2-way mixed-effects model [30]. 

Pearson’s correlation was used to determine correlations between variables; a p-value less than 0.05 was considered statistically significant. In order to identify anatomical parameters useful for Li localization, distances between points measurable by clinical examination were considered independent variables, and distances of the Li from landmarks were considered independent variables. The strengths of correlations were described for the absolute values of the ratios of the compared variables as follows: very weak (0–0.19), weak (0.20–0.39), moderate (0.40–0. 59), strong (0.60–0.79), and very strong (0.80–1.0) [3].

## 3. Results

The study subjects consisted of 111 patients—43 males and 68 females. All results are expressed as the mean of the measured values ± the standard deviation.

The mean age of the patients was 34.93 ± 17.5 years in a range of 18 to 88 years. Results of measurements on CBCT reconstructions are reported in Table 3. The intraexaminer agreement was between good and excellent, depending on which parameter was considered. In the population of the study, the Li was located at 16.96 ± 2.4 mm from the anterior border of mandibular ramus, 15.28 ± 2.1 mm from the posterior border of the ramus, and 13.87 ± 3.69 mm from the mandibular notch. The mean distance of the Li from the distal surface of the mandibular second molar tooth was 29.22 ± 3.98 mm. The vertical distance of the Li from the occlusal plane was 11.22 ± 4.27 mm. The values were higher in men than in women.

We also evaluated the variables, which are dependent and independent on Spix’s spine. 

From Table S1, it is possible to examine the correlations between an independent and a dependent variable. Knowing the value of an independent variable, we have the possibility of hypothesizing the value of a dependent variable. This method is particularly convenient for clinicians who perform block anesthesia to IAN without any certain point of reference. Pearson’s correlation test (Table S1) reported in the study population a very strong correlation between ODLiAB and VDCoMA, ODAPD and VDCoMA, DM2M and VDCoMA, and a strong correlation (COR> 0.60) between ODAPD and ODLiAB, ODLiPB and VDCoMA, VDCoMA and VDLiMB. 

**Figure 2:**
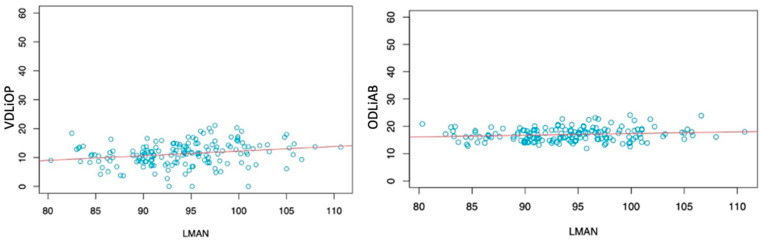
Correlation between LMAN and VDLiOP (**left**) and between LMAN and ODLiAB (**right**).

Statistical analyses showed that LMAN has a statistically significant correlation with VDLiOP and ODLiAB (Figure 2).

## 4. Discussion

Lingula is an important clinical landmark for oral and maxillofacial surgeons. All parameters with the lingula, in relation to various mandibular ramal landmarks, should be considered to avoid intraoperative and post-operative complications.

The inferior alveolar nerve block is the most common technique for providing local anesthesia before restorative and surgical procedures of the mandibular posterior teeth; in certain cases, however, this nerve block fails, even when performed by the most experienced clinician [31].

This high failure rate is often attributed to a high degree of variation in the morphology of the mandibular ramus and the location of the Li [6]. 

The existing measurements using dry human skulls and conventional radiographic techniques have severe limitation: dry human skulls cannot adequately provide the data on sex, age, or race due to lack of information [32].

The Li is visible on radiographs as a well-defined radiopaque image located anteriorly and just superior to the mandibular foramen [33]. Panoramic radiography is not a suitable landmark for the needle insertion point for IAN block due to the distortion present on panoramic radiographs [20,25].

By contrast, measurements of distance and angles on CT and CBCT correspond very closely to the actual size of the object [18]. From studies carried out in the literature, the position of the lingula is influenced by various factors, such as the sex, age, ethnicity, and even the skeletal class of the patient. Different morphological shapes of the lingula were first described by Tuli et al. [34]. The authors classified the morphology of 165 dry adult human mandibles—131 males and 34 females of Indian origin—into 4 types: 1, triangular; 2, truncated; 3, nodular; and 4, assimilated. In most studies, the mandibular lingula is located above the occlusal plane, but occasionally it can be found at or below.

In the present study, Spix’s spine was always located above or at the same height of the occlusal plane; in the study sample, there were no cases with the lingula below the occlusal plane; in both the male and female subjects, there were cases in which Spix’s spine was located at the level of the occlusal plane. In the study sample, the mean of VDLiOP was 34.93 ± 17.46 mm, 11.77 ± 16.68 mm in males and 10.87 ± 16.56 mm in females. 

Jansisyanont et al. [15], in a study carried out on 92 Thai dry skulls, found that in 80.1% of cases, the mandibular lingula was above the occlusal plane, and in 19.9% of them it was below the occlusal plane.

Similarly Sang-Wan et al. [35], in a study on 104 dry mandibles of a Korean population, found that the occlusal plane was between the mandibular foramen and the tip of the lingula in 47.7% of cases, and at the same level as the tip of the lingula in 35.6% of cases.

Zhou et al. [23] found that in 98.3% of cases, Spix’s spine was located superior to the occlusal plane, only in 0.8% of cases was it below the occlusal plane, and in 0.8% it was at the same level of the occlusal plane.

The mean recorded VDLiOP value in the total population of 119 patients was 5.9 ± 3.0 mm, in males it was 6.2 ± 2.8 mm, and in females it was 5.9 ± 3.0 mm. ArunKumar et al. [36], in a study on 100 CBCTs of a South Indian population, observed that the mandibular lingula was, in 31.5% of cases, at the same level as the occlusal plane, in 35.5% above and in 33% positioned below.

Ho-Yeol Jang and Seung-Jung Han, in a study on 125 CBCT images (63 males and 62 females), reported an overall mean for VDLiOP of 8.85 ± 2.59 mm, in males of 10.42 ± 2.52 mm, and in females of 7.42 ± 1.97 mm [37].

The study by Akcay et al. [24], carried out on CBCT images of an adult population aged between 18 and 36 years, observed a mean VDLiOP in the general population of 9.02 ± 3.18 mm, with a maximum of 16.21 mm, and a minimum of 1.84 mm.

Skeletal class can influence the values of VDLiOP: an average of 8.12 ± 2.95 mm was found in patients in the first skeletal class and 9.91 ± 3.16 mm in patients in the third skeletal class.

In the first skeletal class, the mean in males was 8.58 ± 3.20 mm and in females was 7.59 ± 2.59 mm; in the third skeletal class, the mean in males was 9.93 ± 2.44 mm and in females was 9.89 ± 3.72 mm.

Similar results emerge from the study by Sung et al., in which, following an analysis of 60 CT images, the mean VDLiOP was 7.80 ± 2.49 mm in skeletal first class patients and 10.03 ± 3.31 mm in skeletal third class patients [38].

A shortcoming of our study is that the skeletal classes of the patients were not assessed.

Some studies on pediatric patients noted that the distance of Spix’s spine from the occlusal plane increases with age.

Aps et al. [28] studied the position of the mandibular lingula using CBCT data from 280 pediatric patients aged 6 to 18 years. All subjects were classified according to age (6–9 years, 10–13 years, and 14–18 years). The study found that, in 8.9% of cases, the lingula was at or below the occlusal plane. With increasing age, this anatomical feature showed a tendency to decrease regardless of side and gender. The mandibular lingulae positioned to the occlusal planes were higher in the younger group, but relatively unusual in adolescents aged 14 to 18 years.

The minimum distance was recorded in the group of patients aged 6–9 with a VDLiOP of –5.93 mm, the maximum distance was instead observed in the group of patients aged 14–18 with a VDLiOP of 13.65 mm. The approach of this study was also adopted by Krishnamurthy et al., which demonstrated how, through an evaluation of 90 OPT images of patients aged between 7 and 12 years, the distance of the mandibular lingula from the occlusal plane presents a statistically significant gradual increase with increasing age of the patients [16].

Sekerci et al. [6], in a study on CBCT images of 169 pediatric patients (aged 6 to 12 years), showed that in most of the mandibles studied, the mandibular lingula was located above the occlusal plane by 2.0 ± 1.2 mm. In a similar study of 412 CBCT images of pediatric patients carried out by Sekerci, the average was 3.60 ± 1.66 mm [27].

This difference can likely be explained by the different reference points and planes used during the measurement procedure. In the present study, only adults over 18 years of age were included. 

In the Sagittal Split Ramus Osteotomy (SSRO), the lingula is used as a reference point to create the medial osteotomy line: it is therefore important to know the location of the lingula in the anteroposterior width of the mandibular branch [39]. 

In the study population, the mean ODLiAB was 16.96 ± 2.4 mm; in males it was 17.05 ± 2.7 mm and in females it was 16.9 ± 2.19 mm. 

To these values, considering that during anesthesia the needle passes through the soft tissues, it is necessary to add the average thickness of the soft tissues of 4.12 ± 1.21 mm [40], in order to get closer to the mandibular foramen.

Similar results emerge from the literature from the studies by Sekerci and Sisman [27], Soon-Seop et al. [13], and Monnazzi et al. [4]; higher values were found in the study by Kositbowornchai et al. [5] and in the study carried out by Jansisyanont et al. (Table 4) [15]. Only the study by Jang and Han. includes soft tissue thickness in the ODLiAB assessment [37]. 

The studies by Hsu et al. [3] and Kositbowornchai et al. [5] show similar ODLiPB values; lower values emerged in the studies carried out on pediatric patients by Sekerci et al. [6], higher values in the studies done by Zhou et al. [23] and by Jasisyanont et al. [15] (Table 4).

From the studies examined in the literature, it emerges that ODLiAB is always greater than ODLiPB, except in the study by Akcay et al. (Table 4) [24]. From these data, it is possible to deduce that, in the general population, Spix’s spine is located posteriorly with respect to the center of the anteroposterior width of the mandibular branch. By carrying out an evaluation by gender, in males, the mandibular lingula is located almost in the center of the width of the mandibular branch (ODLiAB: 17.05 mm and ODLiPB: 16.04 mm); in females, the lingula is displaced more posteriorly than the center of the mandible (ODLiAB: 16.9 mm and ODLiPB: 14.85). In the study by Zhou et al., ODLiAB and ODLiPB, in males, have the same distance of 18.2 mm, in females, a more posterior location than the center of the anteroposterior width of the mandibular branch mandible was found [23].

In the study population, males have higher ODAPD values than females. Higher values were found in the study by Zhou et al., in which the mean was 33.05 ± 2.4 mm [23].

The mean distance of the lingula to the base of the mandible (VDLiMB) was found to be 31.2 ± 4.35 mm. Similar values were found in Kun-Jung’s studies, with mean values of 31.20 ± 3.81 mm [3]; lower values in the study by ArunKumar et al. [36], with a mean VDLiMB of 14.10 ± 2.5 mm; and higher values emerged in the study by Alves et al. [14].

In the present study, the distance from the lingula to the mandibular notch (VDLiSN), which also helps surgeons to localize the lingula, was found to be 13.87 ± 3.69 mm in the whole sample, 15.01 ± 3.76 mm in males, and 13.34 ± 3.56 mm in females. Females had lower values than males. By making a comparison between VDLiSN and VDLiMB, it is possible to see how Spix’s spine is closer to the sigmoid notch and more distant from the lower edge of the mandible. Different results have emerged from the literature. Sekerci and Sisman [27] found particularly high VDLiSN values with an average of 23.09 ± 3.67 mm; these data are in contrast to the results of ArunKumar et al. [36], in which the mean VDLiSN was 11.4 ± 2.5 mm (Table 4). The average distance of the lingula from the distal aspect of the first molar tooth was found to be 29.22 ±3.98 mm, which was similar to previous studies [15,23,27].

The differences in study design, origin, and age of the subjects could account for the variable results found in other studies. In addition, it is necessary to take into consideration that all subjects have individual anatomical variations. Subjects with elongated and very pointed mandibular lingulae have greater distances from the occlusal planes, while very short lingulae included within the mandibular branch have an equal position of the base of the lingula, and this can lead to a lower value given by the lack of the height of the lingula itself. It is also possible to highlight that in some cases different morphologies of the lingulae on the two mandibular sides may coexist in the same subject. These anatomical variations can explain why there are different data for each study. Moreover, in order to clearly visualize Spix’s spine, the authors adjusted the opacity and brightness of the CBCT images.

In this study, an analysis of the correlation between different measures was carried out, considering some parameters as independent and others as dependent. This was done because the final objective of the study was to identify clinically accessible measures that could allow the localization of Spix’s spine, to allow correct block anesthesia to the inferior alveolar nerve. The results of this study indicate that some clinically accessible anatomical measures are related to the positions of the lingulae; therefore, according to the results of this study, it is possible to obtain a precise method for the localization of the lingula, knowing some anatomical measures of the patient. This result is particularly important because it can clinically be of help in performing block anesthesia to the inferior alveolar nerve. Further studies are needed to confirm the validity of these results.

## 5. Conclusions

The present study provides new information to the literature concerning the location of the mandibular lingula in the Italian population. 

For clinicians, it is essential to be familiar with the anatomy of the Li in order to perform IAN block and orthognathic surgery successfully. The findings of the present study could be utilized in clinical and dental procedures to localize the lingula and avoid intraoperative complications.

Knowledge of the insertion point and of the depth of needle insertion is needed to achieve sufficient anesthesia of the mandible, while morphometric information about the anatomy of the mandible is fundamental to prevent complications in mandibular surgery.

The mean distance of the Li from the distal surface of the mandibular second molar tooth was 29.22 ± 3.98 mm. The vertical distance of the Li from the occlusal plane was 11.22 ± 4.27 mm. 

The results from the present study suggest that clinicians or oral surgeons should insert a needle approximately 16.96 mm from the anterior border of the ramus, and approximately 11.22 mm above the occlusal plane due to fact that the lingulae in the majority of subjects were found above the occlusal plane. In addition, the results help the surgeon evaluate the realization of the osteotomy lines during orthognathic surgery.

## Figures and Tables

**Table 1:** Measures considered in the present study to determine the exact location of the lingula.

Code	Definition	Description
VDLiOP	Distance of the Li from the OP	Measured on the coronal plane and perpendicular to the OP
ODLiAB	Distance of the Li from the anterior border of the mandible parallel to the OP	Measured on the axial plane passing through the ML and parallel to the OP
ODLiPB	Distance of the Li from the posterior border of the ramus	Measured on the axial plane passing through the ML and parallel to the OP
VDLiSN	Distance of the Li from the lower point of the sigmoid notch	Measured on the sagittal plane and perpendicular to the OP
VDLiMB	Distance of the Li from the mandibular base	Measured on the frontal plane passing through the Li and perpendicular to the OP
ODLi2M	Distance of the Li from the distal surface of the mandibular second molar tooth	Measured in the plane parallel to the OP passing through the Li, from the orthogonal projection of the most distal point of the crown to the Li

Li: lingula; OP: occlusal plane.

**Table 2:** Measures between anatomical landmarks to be correlated with the positions of the lingula.

Code	Definition	Description
ODAPD	Anteroposterior diameter of the mandibular ramus	Measured on a plane passing at Li and parallel to the OP.
VDCoMA	Vertical distance between the condyle and mandibular angle	Measured on the sagittal plane as the distance from the highest point of the mandibular condyle to the gonion.
D1M	Mesial–distal diameter of the first mandibular molar	Measured on the sagittal plane as the largest dimension of the crown in the mesial distal direction, parallel to the occlusal surface of the tooth.
D2M	Mesial–distal diameter of the second mandibular molar	Measured on the sagittal plane as the largest dimension of the crown in the mesial distal direction, parallel to the occlusal surface of the tooth.
D3M	Mesial–distal diameter of the third mandibular molar	Measured on the sagittal plane as the largest dimension of the crown in the mesial distal direction, parallel to the occlusal surface of the tooth.
ICD	Intercanine distance	Measured on the frontal plane and parallel to the OP.
DM1M	Distance between the right first mandibular molars and the left ones	Measured on the frontal plane, using the central fossae of the molars as landmarks.
DM2M	Distance between the right second mandibular molars and the left ones	Measured on the frontal plane, using the central fossae of the molars as landmarks.
DM3M	Distance between the right third mandibular molars and the left ones	Measured on the frontal plane, using the central fossae of the molars as landmarks.
DMI	Anteroposterior distance between the intermolar line of the first molars and the midline	Measured on the axial plane as the space between the midline passing through the lower incisors and the intermolar line 36–46.
LMAN	The length of the mandibular body	Measured on the sagittal plane between the most-posterior point of the mandible and the pogonion.

Li: lingula; OP: occlusal plane.

**Table 3:** Results of measurements on CBCT reconstructions. Values are expressed as mean ± standard deviation. ICC: value and 95% confidence interval.

Code	TOTAL (mm)	MALE (mm)	FEMALE (mm)	ICC
Age (years)	34.93 ± 17.46	34.75 ± 18.68	35.07 ± 16.56	
VDLiOP	11.22 ± 4.27	11.77 ± 5.1	10.87 ± 3.61	0.99 (0.98–1.00)
ODLiAB	16.96 ± 2.4	17.05 ± 2.7	16.9 ± 2.19	0.99 (0.97–1.00)
ODLiPB	15.28 ± 2.1	16.04 ± 2.55	14.85 ± 1.65	0.98 (0.95–0.99)
ODAPD	30.51 ± 3.12	31.16 ± 3.68	30.13 ± 2.69	0.99 (0.98–1.00)
VDLiMB	31.2 ± 4.35	33.06 ± 4.4	30.05 ± 3.91	0.99 (0.98–1.00)
ODLi2M	29.22 ± 3.98	30.59 ± 4.01	28.29 ± 3.71	0.99 (0.99–1.00)
VDLiSN	13.87 ± 3.69	15.01 ± 3.76	13.34 ± 3.56	1 (1.00–1)
VDCoMA	55.22 ± 6.58	61.58 ± 5.32	53.1 ± 5.63	0.99 (0.98–1.00)
D1M	10.99 ± 0.7	11.13 ± 0.72	10.89 ± 0.68	1.00 (0.99–1.00)
D2M	10.71 ± 0.9	10.85 ± 0.98	10.61 ± 0.85	0.88 (0.71–0.96)
D3M	10.54 ± 1.04	10.54 ± 1.05	10.54 ± 1.05	0.98 (0.95–0.99)
ICD	27.04 ± 3.16	28.02 ± 3.44	26.46 ± 2.84	0.80 (0.51–0.93)
DM1M	42.18 ± 3.66	43.79 ± 3.22	41.02 ± 3.54	1.00 (0.99–1)
DM2M	48.09 ± 3.42	49.59 ± 3.18	47.01 ± 3.2	0.94 0.87–0.98)
DM3M	54.82 ± 4.95	54.98 ± 5.6	54.66 ± 4.46	0.98 (0.95–0.99)
DMLM	27.97 ± 2.9	28.34 ± 3.34	27.67 ± 2.52	0.86 (0.66–0.95)
LMAN	94.03 ± 5.74	97.28 ± 5.45	92.07 ± 5	0.99 (0.99–1.00)

**Table 4:** Comparison between the results of studies present in the literature and the present study.

Authors	Country	Study Design	Distance (mm)					
			ODLiAB	ODLiPB	VDLiMB	VDLiSN	ODLi2M	ODAPD
Present study (2021)	Italy	CBCT	16.96 ± 2.4	15.28 ± 2.1	31.2 ± 4.35	13.87 ± 3.69	29.22 ± 3.98	30.51 ± 3.12
Sekerci et al. [6] (2013)	Turkey	CBCT	13.3 ± 2.3	10.2 ± 1.6	23.1 ± 3.2	11.4 ± 2.5	24.7 ± 3.7	
Sekerci and Sisman (2013) [27]	Turkey	CBCT	16.77 ± 2.74	13.02 ± 2.31	26.05 ± 3.84	23.09 ± 3.67	29.45 ± 3.92	
Senel et al. [26] (2015)	Turkey	CBCT	18.5 ± 2.3	16.9 ± 3.5	38.3 ± 5.3	18.1 ± 3.6		
ArunKumar et al. [36] (2016)	South India	CBCT	14.05 ± 6.68	12.91 ± 3.73	14.10 ± 4.74	11.4 ± 2.5	16.21 ± 4.85	
Zhou et al. [23] (2017)	Korea	CBCT	18.3 ± 2.3	17.6 ± 1.8	32.9 ± 3.05	15.6 ± 2.5	29.5 ± 3.05	33.05 ± 2.4
Aps et al. [28] (2018)	America	CBCT	18.12 ± 2.17	14.97 ± 1.86	27.38 ± 4.30	15.51 ± 2.36		
Akcay et al. [24] (2019)	Turkey	CBCT	11.6 ± 1.67	16.2 ± 1.76		18.2 ± 2.8		
Jang et al. [37] (2019)	Korea	CBCT	14.68 ± 1.44					
Kun-Jung et al. [3] (2020)	Taiwan	CBCT	19.21 ± 3.02	15.22 ± 2.02	31.20 ± 3.81	20.04 ± 3.16		
Soon-Seop et al. [13] (2002)	Korea	Dry Mandible	16.13 ± 3.53			19.82 ± 5.11		
Kositbowornchai et al. [5] (2007)	Thailand	OPT	23.24 ± 3.82	17.83 ± 3.30				
Kositbowornchai et al. [5] (2007)	Thailand	Dry Mandible	20.7 ± 2.27	15.40 ± 1.90				
Jansisyanont et al. [15] (2009)	Thailand	Dry Mandible	20.6 ± 3.5	18.0 ± 2.6		16.6 ± 2.9	29.7 ± 4.4	
Monnazzi et al. [4] (2011)	Brazil	Dry Mandible	16.5 ± 2.32	14.63 ± 2.13	27.09 ± 5.44	16.38 ± 2.59		
Alves et al. [14] (2015)	Brazil	Dry Mandible	17.76 ± 2.69	15.28 ± 2.31	33.30 ± 4.14	17.29 ± 2.57		

## Supplementary Materials

The following are available online at www.mdpi.com/article/10.3390/healthcare9121747/s1, Table S1: Correlations between dependent and independent variables. The values are represented as: correlation coefficient (95% confidence interval; *p*-value).
